# *Physalis alkekengi* Carotenoidic Extract Inhibitor of Soybean Lipoxygenase-1 Activity

**DOI:** 10.1155/2014/589168

**Published:** 2014-01-09

**Authors:** Veronica Sanda Chedea, Adela Pintea, Andrea Bunea, Cornelia Braicu, Andreea Stanila, Carmen Socaciu

**Affiliations:** ^1^Laboratory of Animal Biology, National Research Development Institute for Animal Biology and Nutrition Baloteşti (IBNA), Calea Bucureşti Nr. 1, Baloteşti, 077015 Ilfov, Romania; ^2^University of Agricultural Sciences and Veterinary Medicine, Cluj-Napoca, 3-5 Manastur Street, 400372 Cluj-Napoca, Romania; ^3^Department of Functional Genomics and Experimental Pathology, Chiricuta Cancer Institute, 400015 Cluj-Napoca, Romania

## Abstract

The aim of this study was to evaluate the effect of the carotenoidic saponified extract of *Physalis alkekengi* sepals (PA) towards the lipoxygenase (LOX) oxidation of linoleic acid. Lipoxygenase activity in the presence of carotenoids, standard and from extract, was followed by its kinetic behaviour determining the changes in absorption at 234 nm. The standard carotenoids used were *β*-carotene (*β*-car), lutein (Lut), and zeaxanthin (Zea). The calculated enzymatic specific activity (ESA) after 600 s of reaction proves that PA carotenoidic extract has inhibitory effect on LOX oxidation of linoleic acid. A longer polyenic chain of carotenoid structure gives a higher ESA during the first reaction seconds. This situation is not available after 600 s of reaction and may be due to a destruction of this structure by cooxidation of carotenoids, besides the classical LOX reaction. The PA carotenoidic extract inhibiting the LOX-1 reaction can be considered a source of lipoxygenase inhibitors.

## 1. Introduction

There are over 600 fully characterized, naturally occurring molecular species belonging to the class of carotenoids. Carotenoid biosynthesis occurs only in bacteria, fungi, and plants where they have established functions that include their role as antenna in the light-harvesting proteins of photosynthesis, their ability to regulate light-energy conversion in photosynthesis, their ability to protect the plant from reactive oxygen species, and coloration [1]. If these were the only known functions/properties of carotenoids in the natural world, continuous research in the field would be adequate; these molecules are also part of the diet in higher species, and in animals and humans, carotenoids assume a completely different set of important function/actions [1].

In humans, some carotenoids (the provitamin A carotenoids: *α*-carotene, *β*-carotene, *γ*-carotene, and the xanthophyll *β*-cryptoxanthin) are best known for converting enzymatically into vitamin A; diseases resulting from vitamin A deficiency remain among the most significant nutritional challenges worldwide [1]. Also, the role that carotenoids play in protecting those tissues that are the most heavily exposed to light (e.g., photo protection of the skin, protection of the central retina) is perhaps most evident, while other potential roles for carotenoids in the prevention of chronic diseases (cancer, cardiovascular disease) are still being investigated [1]. Because carotenoids are widely consumed and their consumption is a modifiable health behaviour (via diets or supplements), health benefits for chronic disease prevention, if real, could be very significant for public health [1].

The existence of an enzyme “carotene oxidase” in soybeans, which catalyzes the oxidative destruction of carotene was reported by Bohn and Haas in 1928 [2]. Four years later, Andre and Hou [2] found that soybeans contained an enzyme, lipoxygenase (linoleate oxygen oxidoreductase), which they termed “*lipoxidase,*” that catalyzed the peroxidation of certain unsaturated fatty acids.

In 1940 the observation that “lipoxidase” is identical to “carotene oxidase” was published [3]. These early findings of lipoxygenase peroxidizing the unsaturated fats and bleaches the carotene were reported as the result of studies on the oxidation of crystalline carotene or carotene dissolved in unsaturated oil. Surprisingly it was found that the carotene oxidase had an almost negligible bleaching action upon the crystalline carotene. On the contrary, when one employs carotene dissolved in a small quantity of fat, the bleaching is extremely rapid. With excessive quantities of fat, the rate of bleaching of the carotene diminishes, and it was concluded that the effect of added fat upon the rate of bleaching of carotene is probably due to a coupled oxidation [3].

Lipoxygenases (EC 1.13.11.12, linoleate:oxygen, oxidoreductases, LOXs), which are found in plants and animals, are a large monomeric protein family with nonheme, nonsulphur, iron cofactor containing dioxygenases catalyzing the oxidation of the polyunsaturated fatty acids (PUFA) as substrate with at least one 1Z,4Z-pentadiene moiety such as linoleate, linolenate, and arachidonate to yield hydroperoxides utilizing Fe^+2^/Fe^+3^ redox potential and molecular oxygen, swapping electrons between intermediate radicals. The enzyme may also catalyze the cooxidation of carotenoids, resulting in the loss of natural colorants and essential nutrients [4].

In pasta the involvement of LOX in color loss is demonstrated by positive correlation between the decrease of *β*-carotene content after pastification and LOX activities in semolina. In addition to this, the hydroperoxidation and bleaching activities of LOX are highly correlated demonstrating that the bleaching might be ascribable to a cooxidative action by LOX, a free-radical-generating biocatalyst [5]. During pasta processing in which the maximal pigment degradation by LOX activity occurs [6], it is shown that externally added *β*-carotene can act as inhibitor of the LOX-catalyzed linoleate hydroperoxidation and an inverse relation between the % of carotenoid loss and the initial carotenoid content in semolina from durum varieties, showing similar LOX activity, was found [7].

Studying the lipoxygenase-catalyzed degradation of carotenoids from tomato Biacs and Daood [8] found that *β*-carotene was the most sensitive component, followed by lycoxanthin and lycopene. Their results also implied that *β*-carotene can actively perform its antioxidant function during the course of lipid oxidation. It seems that oxidative degradation and, accordingly, antioxidant activity of each carotenoid depends on the rate of its interaction with the peroxyl radical produced through the LOX pathway [8] and thus is able to inhibit LOX. The inhibition of the hydroperoxide formation by carotenoids has been attributed to their lipid peroxyl radical-trapping ability [9].

*Physalis alkekengi* (Bladder cherry, Chinese lantern, Japanese lantern, or Winter cherry; Japanese: *hōzuki*) is a relative of *P. peruviana* (Cape Gooseberry), easily identifiable by the larger bright orange to red papery covering over its fruit, which resembles Chinese lanterns. *Physalis alkekengi* varieties are grown for the decorative value of their brilliantly colored, swollen calyces. Its sepals represent rich sources of two important xanthophylls: zeaxanthin and *β*-cryptoxanthin [10, 11]. *β*-cryptoxanthin is one of the xanthophylls with provitamin A activity, a fact that gives it a greater biological importance and application perspectives [10]. Functional role of lutein/zeaxanthin in the human macula, including supporting evidence from epidemiological studies that the higher consumption of these two carotenoids is associated with a lower risk of age-related macular degeneration, makes the areas of photo protection and the potential of prevention of eye diseases by these pigments to continue to be active areas of investigation [1, 12]. The development of 5-LOX inhibitors capable of interrupting the 5-lipoxygenase axis in prostate cancer cells remains the focus of numerous investigations, and there is increasing evidence suggesting that LOX inhibition is a promising therapeutic approach in the treatment of prostate cancer [13, 14]. The polyphenols from *Physalis viscosa* were shown to have anti-inflammatory activity inhibiting 5-LOX [15].

The aim of this study was to evaluate the effect of the carotenoidic saponified extract of *Physalis alkekengi* sepals (PA) towards the LOX-1 oxidation of linoleic acid. Lipoxygenase activity in the presence of carotenoids, standard and from extract, was followed by its kinetic behaviour determining the changes in absorption at 234 nm. The standard carotenoids used were *β*-carotene (*β*-car), lutein (Lut), and zeaxanthin (Zea).

## 2. Materials and Methods

### 2.1. Chemicals

Pure soybean lipoxygenase-1 (LOX-1) was purchased from Sigma Chemical Co., St. Louis, Mo (L-8383), and pure *β*-car from Hoffman la Roche. Linoleic acid (S) and Tween 20 were purchased from Sigma Chemical Co., St. Louis, Mo (L-1376), tetrahydrofuran (THF) super purity grade from Romil Chemicals UK, methanol, ethyl acetate, petroleum ether, and diethyl ether from Merck KGaA, Cluj-Napoca, Romania.

Lut and Zea standard were extracted and purified after a protocol described by Britton et al. [16]. Lut purification was done from Tagetes spp flowers and Zea from *Physalis alkekengi *sepals.

### 2.2. Carotenoid Extraction from *Physalis alkekengi* Sepals

Total carotenoids were extracted from 5 g sepals using a mixture of methanol/ethyl acetate/petroleum ether (1 : 1 : 1, v/v/v) during 4 hours. After filtering the extract, the residue was reextracted two times with the same solvent mixture, following the procedure described by Pintea et al. [10] after Breithaupt and Schwack [17]. The extracts were combined before being partitioned in a separation funnel, successively with diethyl ether, saturated saline solution, and water. The ether phase was evaporated to dryness under vacuum, using a rotary evaporator at 35°C. The evaporated residue (oleoresin) was dissolved in 15 mL of petroleum ether. Half of the oleoresin was dissolved in diethyl ether and saponified overnight, in the dark, at room temperature using 30% methanolic KOH. The saponified extract was washed with saturated saline solution and distilled water, eliminating the soaps and alkaline excess. The organic layer containing carotenoids was dried over anhydrous sodium sulphate and evaporated to dryness.

The carotenoid standards and PA extract were dissolved in diethyl ether and the total carotenoid content was estimated spectrophotometrically. Solutions of 100 *μ*M *β*-car, Lut, and Zea standard and PA extract in THF were prepared in order to assay kinetically their inhibition of LOX-1 activity.

### 2.3. Lipoxygenase Assay and Activity Calculation

The LOX-1 activity for solutions was determined by a modified method of Axelrod et al. [18]. The activity of LOX-1 was determined via the increase in absorbance at 234 nm using a JASCO V-500 spectrophotometer at 25°C as described previously [19] after addition of linoleic acid in borate buffer containing the enzyme. Shortly, in the cuvette containing 0.84 mL 0.2 M borate buffer (pH = 9) and 0.16 mL standard enzyme (1 : 10 containing 46.000 units/mg solid and 63.500 units/mg protein), 0.0084 mL of substrate solution (sodium linoleate 10 mM) were rapidly added and mixed, and the increase in absorbance (*A*) versus the blank was recorded. The blank contained 0.84 mL 0.2 M borate buffer (pH = 9) and 0.16 mL standard enzyme (1 : 10).

The time course of the reaction was registered in each case and the enzymatic specific activity ESA—the variation of the product formation (absorption increase at 234 nm) per time unit and mg enzyme—was determined (AU/sec/mg protein). Each measurement was done in triplicate. For each experimental variant the amount of pure protein taken into reaction was 11.6 × 10^−3 ^mg.

### 2.4. Lipoxygenase Inhibition Assay and Activity Calculation

LOX inhibition assay and activity calculation were performed in the same way like in Section 2.3 but the reaction mixture contained 0.74 mL borate buffer pH = 9, 0.1 mL carotenoid solution 100 *μ*M in THF, 0.16 mL standard enzyme (1 : 10), and 0.0084 mL sodium linoleate 10 mM.

## 3. Results and Discussion

### 3.1. The Kinetic Plot of Standard LOX-1 Reaction in the Absence and in the Presence of Carotenoids (Standard and PA Extract)

**Figure 1:**
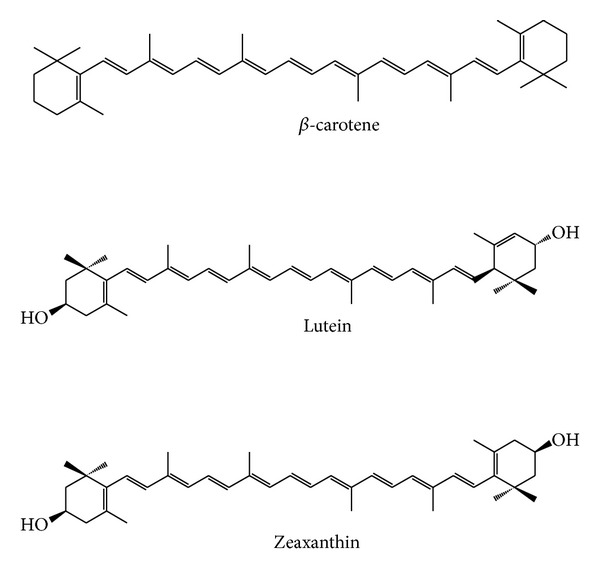
Chemical structures of the standard carotenoids assayed in this study (*β*-car, Lut, and Zea) as LOX-1 inhibitors.

The LOX-1 oxidation of linoleic acid was evaluated in the absence and in the presence of carotenoids. The standard carotenoids were the hydrocarbonic *β*-car and the xanthophylls, Lut and Zea (Figure 1). The PA extract tested was previously analysed by HPLC and contains zeaxanthin and *β*-cryptoxanthin to a ratio of 2.4 : 1 [10]. The UV-Vis measurement wavelength was set at 234 nm in order to register the enzymatic diene conjugation [20].

**Figure 2:**
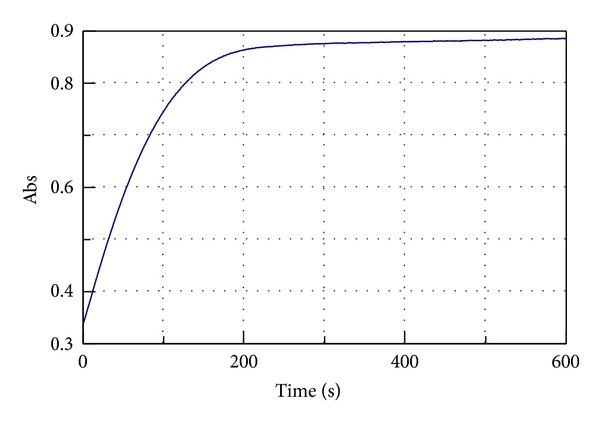
Kinetic plot for pure LOX-1 activity oxidizing the linoleic acid. The absorption (Abs) increase (0–600 sec) at 234 nm indicates the 13-HPOD formation.

Figure 2 shows a typical Michaelis-Menten kinetic plot considered “standard plot” for the hydroperoxides formation (13-HPOD) as reaction products of linoleic acid oxidation by LOX-1. The curve has a “conventional” shape containing an exponential phase followed by a “plateau” phase [19].

**Figure 3:**
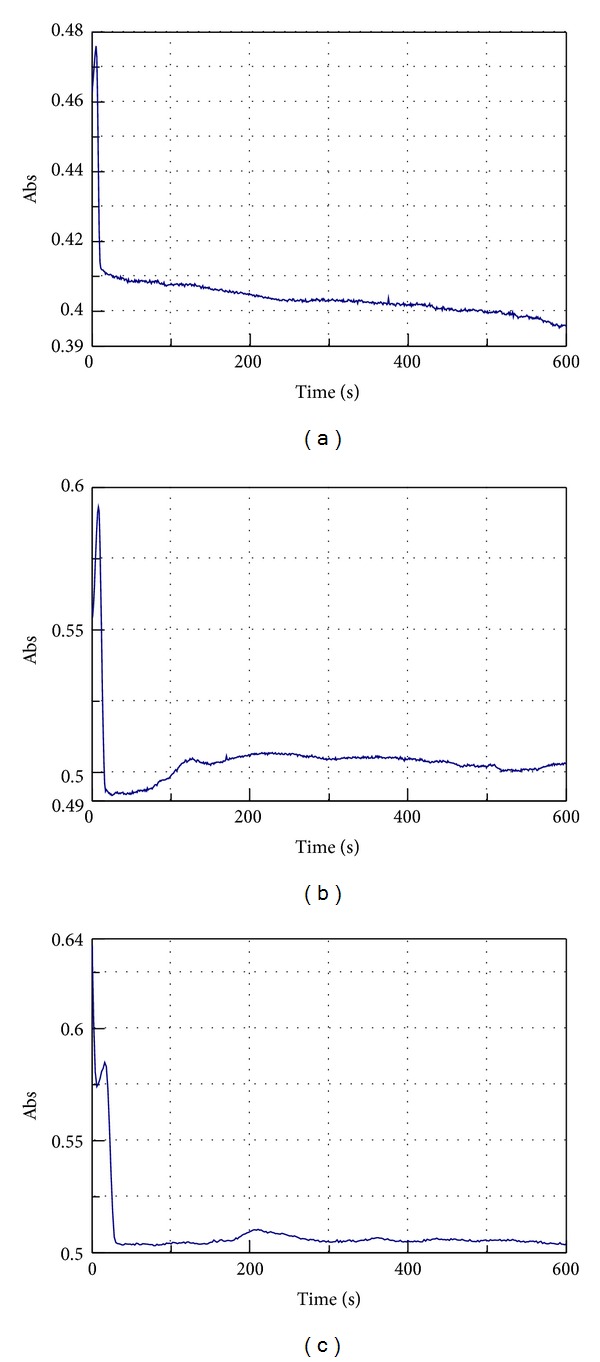
Kinetic plot for pure LOX-1 oxidation of linoleic acid registered at 234 nm in the presence of pure carotenoids: (a) *β*-car, (b) Lut, and (c) Zea.

Adding pure *β*-car, Lut, and Zea to the reaction mixture, the shape of the kinetic plot changes. There are registered three types of curves each of them specific to one carotenoid (Figures 3(a), 3(b), and 3(c)).

Each plot can be divided in two main phases: phase I corresponding to the first 30 seconds of reaction and phase II for the time 30 s–600 s. In function of HPOD formation phase I is subdivided into 2 or 3 other phases.

For *β*-car (Figure 3(a)) and Lut (Figure 3(b)) the first phase of the reaction is alike, characterised by a fast increase followed by a fast decrease. The second phase is represented by a slow decrease for *β*-car (Figure 3(a)) and by a slow increase for Lut (Figure 3(b)). In the case of Zea for phase I the kinetic plot shows a very fast increase during the first 5 seconds then a fast decrease for the next 6 seconds followed again by a slight increase and a fast decrease again. During phase II no enzymatic activity is registered (Figure 3(c)).

The inhibition mechanism of soybean LOX by *β*-car was studied [21]. Addition of *β*-car into the reaction mixture decreased the rate of conjugated diene formation. Increasing the concentration of *β*-car in the reaction mixture resulted in a decrease in the rate of conjugated diene formation [21]. The preferred sites of reaction in a carotenoid molecule are dependent on electron distribution and localization [22]. El-Tinay and Chichester [23] first proposed that the *β*-ionone ring of *β*-carotene was especially prone to attack and that the initial product formed via oxidation would be *β*-carotene 5,6-epoxide [24].

Although Lut and Zea have identical chemical formulas and are isomers, but not stereoisomers, they do not display the same behaviour in the case of LOX oxidation of linoleic acid (Figures 3(b) and 3(c)). Lut and Zea are both polyisoprenoids containing 40 carbon atoms and cyclic structures at each end of their conjugated chains. The main difference between them is in the location of a double bond in one of the end rings giving lutein three chiral centres as opposed to two in zeaxanthin (Figure 1). In membranes it was noted that not all xanthophylls behave the same and small differences in structure alter their behavior, so that, for example, the diols zeaxanthin and lutein orient themselves quite differently in membranes [25]. Such factors would, in turn, be expected to affect their antioxidant ability against carotenoids in the lipid and aqueous phases [24].

**Figure 4:**
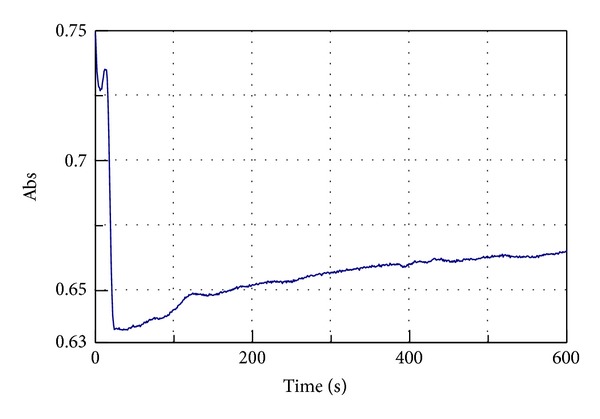
Kinetic plot for pure LOX-1 activity registered at 234 nm in the presence of PA extract.

Figure 4 presents the kinetic of LOX-1 reaction with linoleic acid in the presence of PA.

Phase I has the shape like the one of Zea (Figures 4 and 3(c)) and phase II like Lut (Figures 4 and 3(b)).

### 3.2. LOX-1 Enzymatic Specific Activity (ESA) in the Absence and in Presence of Carotenoids (Standard and PA Extract)

The calculation of the specific enzyme activity was done according to the exponential “burst” phase (I) within the first seconds of reaction (after 5 s) and to the last phase (after 600 s) (Table 1).

Within the first seconds of reaction the highest value of ESA is registered for LOX-1+S+Zea followed by LOX-1+S+PA, LOX-1+S+*β*-car, LOX-1+S+Lut, and at last LOX-1+S.

After 600 s of reaction LOX-1+S has the highest ESA and LOX-1+S+*β*-car the lowest one. LOX-1+S+Lut has the same ESA like LOX-1+S+Zea and lower than LOX-1+S+PA.

The most important factor governing the antioxidant (or even promote prooxidant) activities of carotenoids is its structure (i.e., size, shape, and the nature, position, and number of sustituent groups). It is clear that the structure of a carotenoid molecule effectively dictates how these molecules are incorporated into and may therefore subsequently affect, or control, their local environment [24]. The differences observed in our case, for ESA for LOX-1 activity in absence or presence of carotenoids, are also due to the structural differences between the studied molecules.

Due to the fact that lutein and *β*-carotene have the same shape of the kinetic plot for the first phase of the LOX-1 oxidation of linoleic acid in the presence of carotenoids, we looked at the common features of their chemical structures. Comparing lutein and *β*-carotene it can be seen that the polyenic chain of the 9 conjugated double bonds is the structural element, which is the same for both. The correlation between these common features of the kinetic plots and the common structural element led us to the conclusion that within the first seconds of the oxidation of linoleic acid by LOX-1 there is a modification of the carotenoid structure at the level of the polyene system as already Kennedy and Liebler [26] have shown. The results obtained by Serpen and Gökmen [21] suggest that *β*-carotene reacts with linoleyl radical (L^∙^) at the beginning of the chain reaction, preventing the accumulation of conjugated diene forms (LOO^∙^, LOO^−^, and LOOH). Since L^∙^ transforms back to its original form of LH, the enzyme cannot complete the chain reaction and thus remains at inactive Fe (II) form [21].

The absorption at 234 nm in our case registers the amount of the conjugated double bonds given by the hydroperoxyde formation by LOX-1 oxidation of linoleic acid and also by the polyenic chain of carotenoids so ESA is directly influenced by these two elements.

During phase I of the reaction the calculated ESA for the saponified carotenoids decreases in the order of decreasing the number of double bonds in the polyenic chain from Zea, PA, *β*-car, and Lut. If the polyenic chain is attacked at the 15,15′-double bound as Kennedy and Liebler [26] concluded in the case of lutein less dienic bonds are registered than for *β*-carotene which has 11 double bonds compared to 10 of lutein.

The presence of a hydroxyl group at the 3,3′-position makes the zeaxanthin not to form so effectively the epoxides so the polyenic chain remains intact for longer than *β*-carotene registering at 234 nm a higher absorption and so a higher ESA.

At the end of the second phase of the reaction (after 600 s of reaction) it can be seen that the lowest ESA is given by the LOX reaction in the presence of *β*-carotene showing the fact that *β*-car proves to be the most effective LOX-1 inhibitor, followed by Lut, Zea, and at last PA.

## 4. Conclusions

Conventional chain-breaking antioxidants such as tocopherols trap peroxyl radicals by donating a hydrogen atom. However, *β*-carotene seems to exert an antioxidant activity by a mechanism in which the chain-propagating peroxyl radical is trapped by addition to the conjugated polyene system of *β*-carotene rather than the mechanism of hydrogen donation [9]. The resulting carbon-centered radical is resonance-stabilized because of the delocalization of the unpaired electron in the conjugated polyene system, leading to chain termination. This means that the reaction of *β*-carotene or related carotenoids with the peroxyl radical competes with the production of methyl linoleate hydroperoxides [27].

A cooxidation mechanism is proposed by Wu et al. [28] that involves random attack along the alkene chain of the carotenoid by a LOX-generated linoleoylperoxyl radical.

The decolouring of carotenoids is explained by Jarén-Galán and Mínguez-Mosquera [29] as being due to a loss of conjugation in a sequence of conjugated double bonds. In our case, a longer polyenic chain of carotenoid structure gives a higher ESA during the first reaction seconds. This situation is not available after 600s of reaction and may be due to a destruction of this structure by cooxidation of carotenoids, besides the classical LOX reaction

The PA carotenoidic extract has inhibitory action on LOX-1 so the extract can be considered a source of lipoxygenase inhibitors. It proves that natural extracts could be good candidates for antioxidant action and so, and LOX inhibition like the pure carotenoids lutein, zeaxanthin,  *β*-carotene. Even though more difficult to test, the raw carotenoidic extracts keeping as much as possible the original matrix for antioxidant food supplements could prevent or lower the harmful LOX action in food and human tissues. *Physalis alkekengi* fruit, as a source of zeaxanthin and other carotenoids, would be consumed regularly to complement dietary sources, boosting the amount of these components available from fruits, vegetables, and egg yolks.

## Figures and Tables

**Table 1:** The specific enzyme activities (ESA) (AU/mg/s) for *t*_I_ = 5 s and *t*_II_ = 600 s calculated from the LOX activity plots (Figures 1, 2(a), 2(b), 2(c), and 3) in the absence and presence of carotenoids, pure and in extract.

Exp. var	ESA_I_ × 10^−3^ (AU/mg/s)	ESA_II_ × 10^−3^ (AU/mg/s)
LOX-1 + S	5819	126
LOX-1 + S + Lut	8008	68.9
LOX-1 + S + *β*-car	9976	56
LOX-1 + S + Zea	13724	69
LOX-1 + S + PA	12914	95

S: sodium linoleate; ESA: enzymatic specific activity; AU: absorption units; PA: *Physalis alkekengi* carotenoidic extract; *β*-car: *β*-carotene; Lut: lutein; Zea: zeaxanthin.

## Abbreviations

LOX: Lipoxygenase
9-HPOD: 9-Hydroperoxy-10E,12Z-octadecadienoic acid
13-HPOD: 13-Hydroperoxy-9Z,11E-octadecadienoic acid
S: Sodium linoleate
ESA: Enzymatic specific activity
HPODs: Hydroperoxy-octadecadienoic acids
AU: Absorption units
PA: *Physalis alkekengi* carotenoidic extract
*β*-car: *β*-Carotene
Lut: Lutein
Zea: Zeaxanthin.
